# Label-Free Assay of Protein Kinase A Activity and Inhibition Using a Peptide-Based Electrochemical Sensor

**DOI:** 10.3390/biomedicines9040423

**Published:** 2021-04-13

**Authors:** Hyunju Cho, Chang-Seuk Lee, Tae Hyun Kim

**Affiliations:** 1 Department of ICT Environmental Health System, Graduate School, Soonchunhyang University, Asan 31538, Korea; hyunju98@sch.ac.kr (H.C.); eriklee0329@sch.ac.kr (C.-S.L.); 2 Department of Chemistry, Soonchunhyang University, Asan 31538, Korea

**Keywords:** label-free sensor, peptide electrode, electrochemical sensor, protein kinase A activity, protein kinase A inhibition

## Abstract

We propose a simple label-free electrochemical biosensor for monitoring protein kinase activity and inhibition using a peptide-modified electrode. The biosensor employs cys-kemptide (CLRRASLG) as a substrate peptide which was immobilized on the surface of a gold electrode via the self-assembly of the thiol terminals in cysteine (C) residues. The interaction between protein kinase A (PKA) and adenosine 5′-triphosphate (ATP) on the cys-kemptide immobilized electrode can cause the transfer of ATP terminal phosphates to the peptide substrates at serine (S) residues, which alters the surface charge of the electrode, thus enabling monitoring of the PKA activity via measuring the interfacial electron transfer resistance with electrochemical impedance spectroscopy. The proposed sensor showed reliable, sensitive, and selective detection of PKA activity with a wide dynamic range of 0.1–100 U/mL and a detection limit of 56 mU/mL. The sensor also exhibited high selectivity, rendering it possible to screen PKA inhibitors. Moreover, the sensor can be employed to evaluate the activity and inhibition of PKA in real samples.

## 1. Introduction

Protein phosphorylation by protein kinase is one of the most common and important post-translational modifications, representing the basis of numerous cellular processes, as well as many pathological conditions [1]. Recent research has shown that protein kinases can be implicated in carcinogenesis and the metastasis of several different types of cancers [2]. Thus, protein kinases are known to play a hallmark role in cancers. In addition, a group of researchers has reported that protein kinases can be connected to the infection by the novel severe acute respiratory syndrome coronavirus 2 (SARS-CoV-2) [3]. Therefore, the connection of protein kinases with cancers or COVID-19 allows an insight into the development of potential therapies and drugs that could have therapeutic effects on cancer- or COVID-19-infected patients [2,4].

For drug development and disease diagnosis of such pathological conditions, an effective monitoring system of protein kinase activity and inhibition is crucial to ensure reliable, sensitive, and selective detection. The conventional analytical technique for protein kinase activity is the radioisotopic method utilizing radio-labeled adenosine 5′-triphosphate (ATP), which has the characteristic of being highly sensitive [5]. However, the instrumentation and detection process of the method is time-consuming and expensive. A high risk of radioactive contamination exists when using the radioisotopic method. To overcome the limitations, alternative assays for protein kinase activity have been developed based on many different techniques, including colorimetry [6], mass spectrometry [7], fluorometry [8], flow cytometry [9], resonance light scattering [10], and electrochemical luminescence method [11]. As useful and beneficial as they are, these techniques still have certain drawbacks, such as expensive instrumentation, labor-intensive sampling procedures, and the need for labeling. With its advantages of low production cost, simple instrumentation, fast response, high sensitivity, portability, and label-free detection, the electrochemical methods have attracted the most attention in the assay development of protein kinase activity and inhibition [12]. Furthermore, electrochemical assays are well suited to miniaturization and integration into parallel processing biochips, thereby enabling the use in high-throughput assays [13,14,15].

**Scheme 1:**
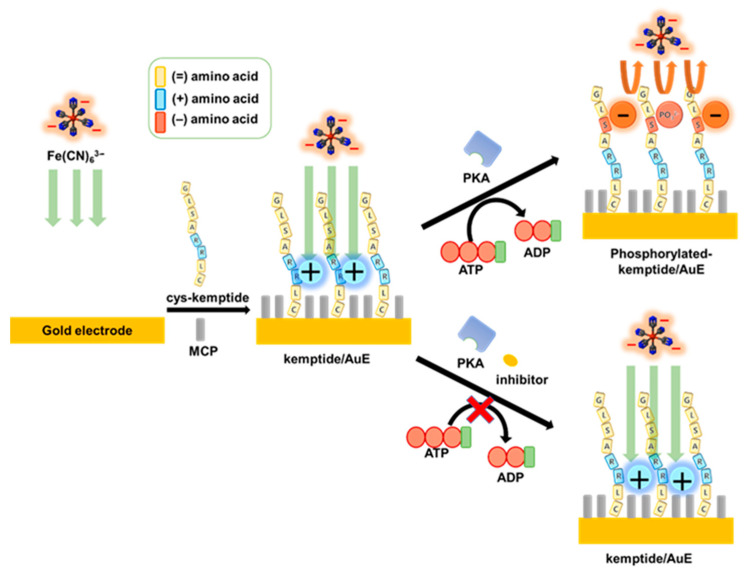
Construction and sensing principle of the peptide-based electrochemical biosensor for the detection of protein kinase A (PKA) activity and inhibition. MCP, 3-mercapto-1-propanol; AuE, Au electrode; ATP, adenosine 5′-triphosphate; ADP, adenosine 5′-diphosphate.

Herein, we report a label-free peptide-based electrochemical biosensor for monitoring the activity and inhibition of protein kinase A (PKA). Scheme 1 depicts the fabrication and sensing principle of the peptide-based biosensor. To construct the biosensor, cysteine-terminated substrate peptides (cys-kemptide, CLRRASLG) were immobilized on the surface of a gold electrode via gold-thiol self-assembly. When the target PKA is added to the sensing system in the presence of adenosine 5′-triphosphate (ATP) as the co-substrate (as the phosphate group donor), the site-specific phosphorylation would occur on serine (S) residues in the cys-kemptide-immobilized gold electrode (kemptide/AuE). This phosphorylation makes the electrode surface negatively charged, which can interrupt the penetration of the external redox probe ((Fe(CN)_6_)^3−^) into the Au electrode surface, thus resulting in the hindered electron transfer of the redox probe and the increase of the impedance in the system. On the other hand, the addition of an inhibitor in the sensing system makes it difficult for PKA to regulate the phosphorylation on the modified electrode, resulting in no change in electrochemical response, thus enabling it to be employed for the identification of new PKA inhibitors. With the advantages of the simple fabrication method and the label-free sensor, as well as sensitive detection of electrochemical impedance spectroscopy (EIS), the proposed biosensor has been successfully applied to the monitoring of PKA activity and inhibition with satisfactory results. Therefore, the developed biosensor shows great promise for clinical application and pharmaceutical research.

## 2. Materials and Methods

### 2.1. Chemicals

Protein kinase A (PKA), ATP, N-(2-(p-bromocinnamylamino)ethyl)-5-iso-quinolinesulfonamide dihydrochloride (H-89 dihydrochloride hydrate), 3-mercapto-1-propanol (MCP), phosphate- buffered saline (PBS, pH 7.4, 0.01 M), potassium ferricyanide (K_3_Fe(CN)_6_), human serum albumin (HSA), thrombin, haemoglobin (Hb), and glucose were obtained from Sigma-Aldrich Co., (St. Louis, MO, USA). cys-kemptide (H-CLRRASLG-NH_2_) was synthesized by Peptron Co., (Daejeon, Korea). Deionized (DI) water was prepared in a Millipore water purification system (Milli-Q, specific resistivity >18 MΩ cm, Millipore Korea, Co., Ltd.; Seoul, Korea).

### 2.2. Preparation of the Peptide-Based Sensor

Before the modification of the electrode, a 3 mm diameter bare Au disk electrode was polished with 1, 0.3, and 0.05 μm Al_2_O_3_ powders, respectively. Then, to remove the residual powder, the electrode was cleaned by using a mixed solution of ethanol and DI water (1:1, ethanol: DI water) in an ultrasonic bath for 10 min. Afterward, the surface of the Au electrode was electrochemically activated in 0.5 M H_2_SO_4_ solution by cyclic voltammetry (CV) cycling between 0.2 and 1.6 V at a scan rate of 0.1 V s^−1^ until a steady reproducible CV curve was obtained. To immobilize cys-kemptide on the Au electrode, 10 μL of 0.5 mM cys-kemptide solution (in 10 mM pH 7.4 PBS solution) solution was dropped on the electrode and incubated in humid conditions for 30 min. To passivate the regions between the peptide strands on the modified electrode, 10 μL of 10 mM MCP solution was drop-casted and incubated for 3 h in humid conditions, followed by DI water wash and N_2_ dry.

### 2.3. Electrochemical Measurements

All electrochemical experiments were performed with a Model 660D electrochemical analyzer (CH Instruments, Inc., Austin, TX, USA) using a conventional three-electrode cell. Bare Au and kemptide/Au electrodes were used as working electrodes. A Pt wire and an Ag/AgCl (3 M NaCl) electrode were used as a counter and a reference electrode, respectively. EIS studies were carried out in 0.01 M PBS buffer (pH 7.4) containing 2 mM K_3_Fe(CN)_6_. EIS spectra were recorded in the frequency range of 10^5^–10^−2^ Hz at a sinusoidal voltage perturbation of 5 mV amplitude. To evaluate ATP-dependent PKA activity, various concentrations of ATP were tested in 0.01 M PBS containing 2 mM K_3_Fe(CN)_6_ and 100 U/mL PKA on kemptide/AuE after incubation for 1 h. For PKA activity measurements, various concentrations of PKA were tested with 10 μM ATP in 0.01 M PBS containing 2 mM K_3_Fe(CN)_6_ on kemptide/AuE after incubation for 1 h.

For the PKA inhibition test, various concentrations of protein kinase inhibitor H-89 were initially incubated with 100 U/mL PKA and 10 μM ATP for 1 h, and then the activity of PKA was measured. For selectivity experiments, 10 mg/mL of potential interferences, such as HSA, thrombin, Hb, and glucose, were tested with 10 μM ATP in 0.01 M PBS containing 2 mM K_3_Fe(CN)_6_ on kemptide/AuE after incubation for 1 h. For real sample analysis of PKA activity and inhibition, HeLa cell lysates containing PKA were tested in 0.01 M PBS containing 10 μM ATP and 2 mM K_3_Fe(CN)_6_ on kemptide/AuE in the presence and the absence of 50 μM H-89, after the incubation for 10 min and 60 min.

### 2.4. Preparation of HeLa Cell Lysates

For the real sample analysis, the human cervical cancer cell line HeLa was obtained from the Korean Cell Line Bank (Seoul, Korea). HeLa cells were cultured in Roswell Park Memorial Institute (RPMI) 1640 media containing 10% FBS and 1% ABS at 37 °C in an incubator containing 5% CO_2_. The cultured HeLa cells were washed in Dulbecco’s Phosphate-Buffered Saline (DPBS) and lysed in Pro-PREP protein extraction solution (INTRON, Seongnam, Korea) at 4 °C for 8 h. The lysate was centrifuged at 4 °C, 15,000 rpm, for 15 min, and the supernatant was boiling to determine. Then, the obtained supernatants were transferred to a freezing centrifuge tube (Axygen, Union City, CA, USA) and stored at −20 °C in a refrigerator. The concentration of protein was determined by the bicinchoninic acid (BCA) assay using a Microplate Absorbance Spectrophotometer at 562 nm. All cell lysates were diluted to 10 μg/mL total protein concentration for kinase activity assay.

## 3. Results and Discussion

### 3.1. Fabrication and Feasibility of the Biosensor

**Figure 1:**
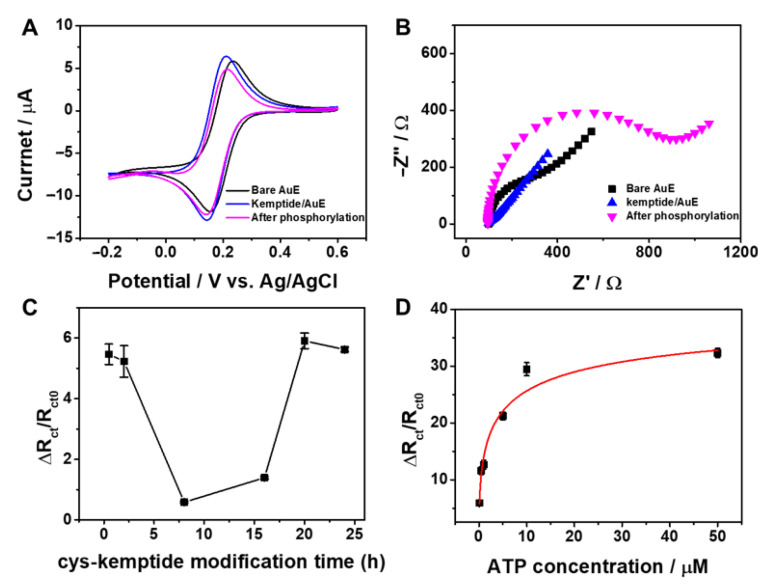
(**A**) CV and (**B**) EIS responses of bare Au electrode and kemptide/AuE before and after phosphorylation by 100 U/mL PKA in 0.01 M phosphate-buffered saline (PBS) solution (pH 7.4) containing 10 μM adenosine 5′-triphosphate (ATP) and 2 mM K_3_Fe(CN)_6_ redox probe. (**C**) The effect of cys-kemptide modification time (0.5, 2, 8, 16, 20, 24 h) on the change in the electrochemical responses before and after the phosphorylation by 100 U/mL PKA in 0.01 M PBS solution (pH 7.4) containing 10 μM ATP and 2 mM K_3_Fe(CN)_6_. (**D**) The effect of ATP concentration (0.1, 0.5, 1, 5, 10, 50 μM) on the change in the electrochemical responses before and after the phosphorylation by 100 U/mL PKA in 0.01 M PBS solution (pH 7.4) containing 2 mM K_3_Fe(CN)_6_. Each experiment was performed in triplicate.

Cyclic voltammetry (CV) and EIS experiments were employed to study the fabrication process and the feasibility of the proposed sensor by using (Fe(CN)_6_)^3−^ as a redox probe. Figure 1A shows CV curves for a 2 mM solution of (Fe(CN)_6_)^3−^ in 10 mM PBS (pH 7.4) recorded with a bare Au electrode, following modification with a mixed layer of cys-kemptide and MCP, and subsequent phosphorylation of kemptide on the Au electrode by PKA in the presence of ATP. All three electrodes showed a pair of well-defined redox peaks. However, peak-to-peak separation, ΔE_p_ (67 mV) for kemptide/AuE is smaller than that for bare Au electrode (86 mV), while the redox peak currents, i_p_s for kemptide/AuE are bigger. This is because two arginine (R) residues at the internal position in kemptide are charged positively [16] in pH 7.4 on the surface of Au electrode, which results in the attractive interaction between negative Fe(CN)_6_^3−^ and the electrode, leading to a relatively facile diffusion process of redox probe to the electrode surface (Scheme 1). After the addition of PKA and ATP into the system, the shape of the CV curve changed with increased ΔE_p_ and decreased i_p_s, indicating that serine residue (S) at the upper internal position in kemptide was phosphorylated on the Au electrode surface, which caused the repulsive effect of positively charged kemptide on the electron transfer of the (Fe(CN)_6_)^3−^ to the electrode surface (Scheme 1). These results clearly show the proposed kemptide/AuE biosensor was successfully fabricated and could be used for monitoring the PKA activity.

Electrochemical impedance spectroscopy (EIS) measurements also confirm the fabrication and the feasibility of the proposed biosensor. EIS results are presented using Nyquist plots comprising of a semicircle in the high-frequency region and a slope in the low-frequency region, as shown in Figure 1B. Charge transfer resistance, ΔR_ct_ estimated from the semicircle diameter is the most sensitive and immediate parameter to reflect the changes of the electrode interface. As shown in Figure 1B, compared to the bare Au electrode, the electrode after self-assembly of cys-kemptide exhibits a smaller semicircle domain with an ΔR_ct_ of 22.4 Ω, indicating a very low electron transfer resistance to the redox probe in the electrolyte solution. This is ascribed to the attractive effect of positively charged arginine residues in the kemptide on the redox probe, verifying the immobilization of cys-kemptide on the Au electrodes surface, thus forming the proposed kemptide/AuE biosensor. After the introduction of PKA and ATP into the system, the diameter of the semicircle increased dramatically and the ΔR_ct_ was about 709 Ω due to the phosphorylation of serine residue at kemptide/AuE and the subsequent repulsion between (Fe(CN)_6_)^3−^ and the negatively charged electrode surface. This result verifies the sensing principle of the proposed kemptide/AuE biosensor which can be employed for the label-free electrochemical detection of PKA activity by CV and EIS experiments. However, as shown in Figure 1A,B, the EIS experiments exhibited more sensitive measurements, thus the following measurements have been performed by impedimetric experiments.

The peptide immobilization condition is an essential factor to fabricate a biosensor with good performance, therefore it was optimized by controlling the immobilization time before the impedimetric analysis. The Au electrodes were separately incubated with 0.5 mM cys-kemptide solution for the different time from 30 min to 24 h, and then their ΔR_ct_ change before and after the phosphorylation was compared. The ΔR_ct_ change was estimated by the equation, ΔR_ct_/R_ct0_ where ΔR_ct_ is (R_ct_–R_ct0_), and R_ct0_ and ΔR_ct_ are electron transfer resistances before and after the phosphorylation. As shown in Figure 1C, the ΔR_ct_/R_ct0_ value decreases with the increase of immobilization time from 30 min to 6 h, and then it increases until 20 h. Although the ΔR_ct_/R_ct0_ values for 30 min and 20 h are comparable, 30 min was set as the optimal time for peptide immobilization in a time-effective manner.

ATP plays a critical role as a co-substrate in the PKA-regulated phosphorylation of peptides or proteins, which provides phosphate groups for the whole process. Hence, the effect of ATP concentrations on the electrochemical response was examined. Figure 1D shows the electrochemical responses with increasing concentrations of ATP from 0.1 μM to 50 μM in the presence of 100 U/mL PKA. The ΔR_ct_/R_ct0_ value gradually increased with increasing concentration of ATP and saturated after the concentration reached 10 μM. Therefore, 10 μM ATP was used in the phosphorylation reaction. The Michaelis–Menten constant, K_m_ of PKA for ATP, could be calculated to be 7.4 × 10^−7^ M from the ATP titration curve in Figure 1D.

### 3.2. Analytical Performance toward PKA Activity and Inhibition

**Figure 2:**
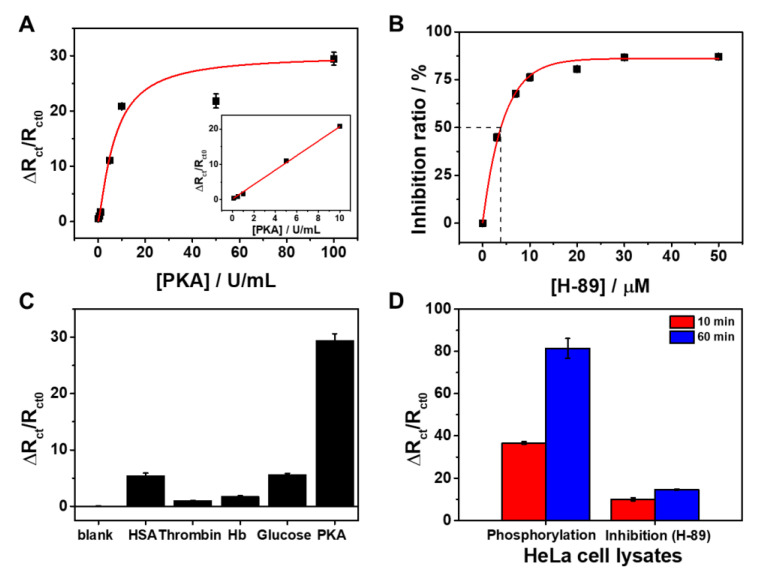
(**A**) ΔR_ct_ change responses of kemptide/AuE upon the addition of increasing concentration of PKA (0.01, 0.05, 0.1, 0.5, 1, 5, 10, 50, 100 U/mL). (**B**) The inhibition effect of different concentrations of H-89 on the response of kemptide/AuE. (**C**) The interference test of human serum albumin (HSA), thrombin, haemoglobin (Hb), and glucose on the determination of PKA. The PKA concentration was 100 U/mL while other species were 10 mg/mL. (**D**) PKA activity and inhibition assay of the sensor in HeLa cell lysates at incubation time of 10 min (red bars) and 60 min (blue bars) in the absence (left bars) and presence (right bars) of 50 μM H-89. Each experiment was performed in triplicate.

Under optimal conditions, the analytical performance of the proposed biosensor was investigated with different concentrations of PKA. Figure 2A shows ΔR_ct_ change responses of the kemptide/AuE sensor upon the addition of increasing concentration of PKA from 0.1 U/mL to 100 U/mL. The ΔR_ct_ change response of the kemptide/AuE sensor increased with increasing concentrations of PKA from 0.1 U/mL to 10 U/mL, afterward the response was saturated. The linear range is represented by the linear regression equation: ΔR_ct_/R_ct0_ = 2.05 (PKA) (U/mL) + 0.241 (*r*^2^  =  0.986). The limit of detection (LOD) was calculated to be 0.056 U/mL (S/N = 3), which is comparable to those of the previously reported sensitive methods as summarized in Table 1. Along with its attractive features, such as simple fabrication and facile operational convenience, the proposed sensor is highly applicable to the sensitive monitoring of protein kinase activity with a wide dynamic range. We further evaluated the applicability of the kemptide/AuE sensor in screening potential protein kinase inhibitor. As a model inhibitor, a synthesized highly selective inhibitor, H-89, was chosen to study the PKA inhibition assay. The inhibition study was performed with different concentrations of H-89 from 3 μM to 50 μM in the presence of 100 U/mL PKA and 10 μM ATP, and the inhibition effect was estimated from the following equation:

(1) $$\mathrm{Inhibition}\;\mathrm{ratio}\;\left(\%\right)=\;\left(1-\frac{{\Delta R}_{\mathrm{ct}}\;\mathrm{change}\;\mathrm{with}\;H-89}{{\Delta R}_{\mathrm{ct}}\;\mathrm{change}\;\mathrm{without}\;H-89}\right)\times 100$$

As shown Figure 2B, the inhibition ratio increased correspondingly with increasing concentrations of H-89 and reached the minimum value at about 10 μM. The half-maximal inhibitory value (IC_50_) was calculated to be about 3.85 μM (Figure 2B, curve a), which is comparable to the previously reported values [11,17]. This result clearly demonstrates that the proposed biosensor can be employed for the screening of protein kinase inhibitors and thus is promising for its potential application in drug discovery and disease therapy. To examine its specificity for the analysis of PKA activity, the proposed biosensor was challenged with potential interferences, such as HSA, thrombin, Hb, and glucose. These biomolecules are possibly present together as a mixture in biological fluids, representing a complex biological matrix. As shown in Figure 2C, the addition of the interferents caused little changes in the impedimetric signal relative to the blank control, while the addition of PKA led to a noticeable ΔR_ct_ change response. This proves that the proposed biosensor has high selectivity toward PKA and can be utilized for analysis of complex real samples. Indeed, we could detect PKA activity and inhibition in complex biological samples, such as cell lysates. Figure 2D shows PKA activity and inhibition assay of the sensor in HeLa cell lysates. As shown in the left bars of Figure 2D, the electrochemical response increased with the incubation time, which may reflect a time-dependent rise in PKA activity. In contrast, when H-89 was introduced into the cell lysate, the sensor responses were suppressed, indicating H-89 inhibited the enzymatic activity of PKA (Figure 2D right bars).

## 4. Conclusions

We have proposed a facile and label-free assay to detect PKA activity and inhibition using a peptide electrode-based electrochemical biosensor. The proposed biosensor has shown a good sensitivity with a wide dynamic range and a high selectivity owing to the specific catalytic ability of PKA towards its substrate peptide. Furthermore, the biosensor has demonstrated great potential in kinase activity monitoring and its inhibitor screening in complex real samples. The electrochemical assay reported here provides a powerful platform for monitoring protein kinase activities and promises to spur the development of new protein kinase inhibitors. This is a prominent advance since many protein kinases are involved in numerous forms of cancer and other diseases, and are thus considered immediate therapeutic targets. If this electrochemical assay is adapted in a microchip format, it will be possible to achieve high-throughput screening for drug discovery and protein kinase profiling of patient samples.

## Figures and Tables

**Table 1:** The comparison of the sensing performance of the proposed biosensor with other methods for PKA.

Method	Linear Range (U/mL)	LOD (U/mL)	Refs.
Electrochemiluminescence	0.05–100	0.17	[11]
Electrochemiluminescence	0.07–32	0.07	[18]
Electrochemiluminescence	0.1–10	0.09	[19]
Fluorimetry	1–2000	0.47	[20]
Fluorimetry	0.1–6	0.038	[21]
Fluorimetry	0.05–1.6	0.02	[22]
Fluorimetry	0.1–1	0.03	[23]
Fluorimetry	0.1–2000	0.039	[24]
Colorimetry	0–1.0	0.232	[25]
Electrochemistry	0.1–10	0.056	This work

## Data Availability

The data presented in this study are included in this published article.

## References

[1] Tarrant M.K., Cole P.A. (2009). The Chemical Biology of Protein Phosphorylation. Annu. Rev. Biochem..

[2] Bhullar K.S., Lagarón N.O., McGowan E.M., Parmar I., Jha A., Hubbard B.P., Rupasinghe H.P.V. (2018). Kinase-Targeted Cancer Therapies: Progress, Challenges and Future Directions. Mol. Cancer.

[3] Bouhaddou M., Memon D., Meyer B., White K.M., Rezelj V.V., Marrero M.C., Polacco B.J., Melnyk J.E., Ulferts S., Kaake R.M. (2020). The Global Phosphorylation Landscape of SARS-CoV-2 Infection. Cell.

[4] Ardito F., Giuliani M., Perrone D., Troiano G., Muzio L.L. (2017). The Crucial Role of Protein Phosphorylation in Cell Signaling and its Use as Targeted Therapy (Review). Int. J. Mol. Med..

[5] Hastie C.J., Mclauchlan H.J., Cohen P. (2006). Assay of Protein Kinases Using Radiolabeled ATP: A Protocol. Nat. Protoc..

[6] Wei H., Chen C., Han B., Wang E. (2008). Enzyme Colorimetric Assay Using Unmodified Silver Nanoparticles. Anal. Chem..

[7] Deng Z., Ye M., Bian Y., Liu Z., Liu F., Wang C., Qin H., Zou H. (2014). Multiplex Isotope Dimethyl Labeling of Substrate Peptides for High Throughput Kinase Activity Assay via Quantitative MALDI MS. Chem. Commun..

[8] Jia C., Bai J., Liu Z., Gao S., Han Y., Yan H. (2020). Application of a Titanium-Based Metal-Organic Framework to Protein Kinase Activity Detection and Inhibitor Screening. Anal. Chim. Acta.

[9] Ren W., Liu C., Lian S., Li Z. (2013). Flow Cytometry-Assisted Mix-and-Read Assay for Ultrasensitive Detection of Protein Kinase Activity by Use of Zr4+-Functionalized Mesoporous SiO2 Microspheres. Anal. Chem..

[10] Li T., Liu X., Liu D., Wang Z. (2013). Sensitive Detection of Protein Kinase A Activity in Cell Lysates by Peptide Microarray-Based Assay. Anal. Chem..

[11] Luo Q.-X., Li Y., Liang R.-P., Cao S.-P., Jin H.-J., Qiu J.-D. (2020). Gold Nanoclusters Enhanced Electrochemiluminescence of g-C3N4 for Protein Kinase Activity Analysis and Inhibition. J. Electroanal. Chem..

[12] Tan D., Li F., Zhou B. (2019). Electrochemical Assay Methods for Protein Kinase Activity. Int. J. Electrochem. Sci..

[13] Nemčeková K., Labuda J. (2021). Advanced Materials-Integrated Electrochemical Sensors as Promising Medical Diagnostics Tools: A Review. Mater. Sci. Eng. C.

[14] Tu J., Torrente-Rodríguez R.M., Wang M., Gao W. (2020). The Era of Digital Health: A Review of Portable and Wearable Affinity Biosensors. Adv. Funct. Mater..

[15] Fernández-La-Villa A., Pozo-Ayuso D.F., Castaño-Álvarez M. (2019). Microfluidics and Electrochemistry: An Emerging Tandem for Next-Generation Analytical Microsystems. Curr. Opin. Electrochem..

[16] Harms M.J., Schlessman J.L., Sue G.R., Garcia-Moreno B. (2011). Arginine Residues at Internal Positions in a Protein are Always Charged. Proc. Natl. Acad. Sci. USA.

[17] Yan Z., Wang Z., Miao Z., Liu Y. (2016). Dye-Sensitized and Localized Surface Plasmon Resonance Enhanced Visible-Light Photoelectrochemical Biosensors for Highly Sensitive Analysis of Protein Kinase Activity. Anal. Chem..

[18] Xu S., Liu Y., Wang T., Li J. (2010). Highly Sensitive Electrogenerated Chemiluminescence Biosensor in Profiling Protein Kinase Activity and Inhibition Using Gold Nanoparticle as Signal Transduction Probes. Anal. Chem..

[19] Wang Z., Yan Z., Sun N., Liu Y. (2015). Multiple Signal Amplification Electrogenerated Chemiluminescence Biosensors for Sensitive Protein Kinase Activity Analysis and Inhibition. Biosens. Bioelectron..

[20] Shen C., Xia X., Hu S., Yang M., Wang J. (2015). Silver Nanoclusters-Based Fluorescence Assay of Protein Kinase Activity and Inhibition. Anal. Chem..

[21] Wang M., Wang L., Liu Q., Su X. (2018). A Fluorescence Sensor for Protein Kinase Activity Detection Based on Gold Nanoparticles/Copper Nanoclusters System. Sens. Actuators B Chem..

[22] Liu Q., Na W., Wang L., Su X. (2017). Gold Nanocluster-Based Fluorescent Assay for Label-Free Detection of Protein Kinase and its Inhibitors. Microchim. Acta.

[23] Zhou J., Xu X., Liu W., Liu X., Nie Z., Qing M., Nie L., Yao S. (2013). Graphene Oxide–Peptide Nanocomplex as a Versatile Flu-orescence Probe of Protein Kinase Activity Based on Phosphorylation Protection against Carboxypeptidase Digestion. Anal. Chem..

[24] Wang L., Yan X., Su X. (2016). A Label-Free and Sensitive Fluorescent Assay for One Step Detection of Protein Kinase Activity and Inhibition. Anal. Chim. Acta.

[25] Zhou J., Xu X., Liu X., Li H., Nie Z., Qing M., Huang Y., Yao S. (2014). A Gold Nanoparticles Colorimetric Assay for Label-Free Detection of Protein Kinase Activity Based on Phosphorylation Protection Against Exopeptidase Cleavage. Biosens. Bioelectron..

